# Characterization of Composites from Post-Consumer Polypropylene and Oilseed Pomace Fillers

**DOI:** 10.3390/polym16243557

**Published:** 2024-12-20

**Authors:** Karolina Lipska, Izabela Betlej, Katarzyna Rybak, Małgorzata Nowacka, Piotr Boruszewski

**Affiliations:** 1 Department of Technology and Entrepreneurship in Wood Industry, Institute of Wood Sciences and Furniture, Warsaw University of Life Sciences—SGGW, 159 Nowoursynowska St., 02-776 Warsaw, Poland; karolina_lipska@sggw.edu.pl; 2 Department of Wood Science and Wood Protection, Institute of Wood Sciences and Furniture, Warsaw University of Life Sciences—SGGW, 159 Nowoursynowska St., 02-776 Warsaw, Poland; izabela_betlej@sggw.edu.pl; 3 Department of Food Engineering and Process Management, Institute of Food Sciences, Warsaw University of Life Science—SGGW, 159C Nowoursynowska St., 02-776 Warsaw, Poland; katarzyna_rybak@sggw.edu.pl

**Keywords:** biocomposites, plant waste, natural fibers, polypropylene, thermoplastics

## Abstract

This study investigates the properties of composites produced using post-consumer polypropylene (PP) reinforced with lignocellulosic fillers from *Nigella sativa* (black cumin) and rapeseed pomace. Using agri-food by-products like pomace supports waste management efforts and reduces the demand for wood in wood–plastic composites. The composite production method combined extrusion and hot flat pressing. Mechanical tests showed a decrease in the tested parameters. Compared to the control variant, the MOE decreased by 26.4% (PP_R variant) and 46.9% (PP_N variant), and the MOR value decreased by 78.7% (PP_N) and 55.1% (PP_R). No significant differences in surface roughness parameters were observed. The composite with nigella particles demonstrated increased wettability. TGA tests showed reduced thermal stability compared to PP and differences between composite variants. The composites exhibited susceptibility to fungal overgrowth, which suggests potential biodegradability. The composites demonstrated complete overgrowth by inoculated fungi, reaching 100% coverage, while samples from PP known to be resistant to biological factors remained unaffected. Although the mechanical properties of the composites were degraded, the use of lignocellulosic fillers offers undeniable advantages, such as waste management of lignocellulosic and polypropylene byproducts, reduced wood demand, and the potential biodegradability of the obtained composites. However, there is a need for further optimization of manufacturing processes and material composition to enhance the material performance.

## 1. Introduction

Biodegradable polymers are an important group of materials capable of degradation due to biological processes, making them extremely important in environmental protection, especially in the face of the growing problem of microplastic pollution. The development of biodegradable polymer production technologies is becoming increasingly advanced. This allows for the creation of materials with appropriate durability and favorable utility properties, making them an attractive alternative to traditional plastics. An example of sustainable technologies in the production of plastics is introducing a plant component into the polymer matrix.

Polymer materials based on petroleum-derived raw materials with lignocellulosic components are widely described in the literature [1,2,3,4]. The plant component is biodegradable and acts as a reinforcement in polymer matrices. Adding plant particles to a composite based on a matrix of popular petroleum-derived polymers can give new properties and functions and make the composite more environmentally friendly [5]. Many studies have highlighted that incorporating natural fillers into a polymer matrix enhances composite biodegradability. For example, Ray and Das [6] demonstrated that polypropylene composites reinforced with Bauhinia Vahlii Fiber exhibited enhanced biodegradability under soil burial and natural weathering conditions. Similarly, studies by Nourbakhsh et al. [7] have indicated that the addition of rice-husk fiber and bagasse fiber to polypropylene composites improved their biodegradation in soil burial tests.

Polypropylene (PP), along with polyethylene, is a thermoplastic produced on a large scale and in large quantities. To improve its properties and give it new functions, researchers have conducted numerous tests involving the reinforcement of polypropylene with plant particles. Agave [8], olive pits [9], rice husks [10], and sisal [11] have already been used in this respect. Naghmouchi et al. [12] showed that adding wood flour as a reinforcing element of PP improves the tensile strength of the resulting composite. Another interesting solution may be the addition of a filler to PP in the form of walnut shell flour. Such a filler improves the hardness and stiffness modulus of the composite but reduces the tensile strength [13]. Improving mechanical and physical properties does not depend solely on the filler. Compatibility between polar (polypropylene) and non-polar (lignocellulosic particles) components is important and can be enhanced through chemical modification, thus improving the final properties of the composite [14].

Food production waste can become a promising raw material in producing new-generation composites. Raw food materials are also a rich source of substances, the presence of which can significantly contribute to improving the functional properties of composites produced with their participation or giving completely new features. Polyphenols, alkaloids, or carboxylic acids present in particles of natural origin incorporated into the polymer matrix cause the composite to have antioxidant [15] and antibacterial [16] properties, thanks to which its application can be extended to those areas where biological properties are desired.

The aim of this study is to investigate the potential of composites derived from post-consumer polypropylene (PP) reinforced with oilseed pomace fillers, specifically from Nigella sativa (black cumin) and rapeseed. With the growing problem of plastic waste and agri-food residues, as well as the increasing demand for environmentally friendly materials, this study focuses on reducing waste and developing innovative materials by using easily available post-consumer plastics and agricultural byproducts. The use of agricultural by-products, such as black cumin and rapeseed pomace, contributes to waste reduction and the circular economy by utilizing waste materials instead of virgin resources. Using post-consumer and waste materials is economically advantageous due to their typically lower cost compared to primary raw materials. Additionally, processing waste helps reduce costs associated with their storage and disposal. As a result, production processes become more cost-effective and environmentally friendly. In the case of oilseed pomace, its advantage lies in the fact that there is no need for collection from the field or extraction. Its form makes it easy to store and transport. The use of lignocellulosic particles of non-wood origin offers the possibility to reduce the consumption of wood raw materials, contributing to forest protection and promoting sustainable material sourcing.

An essential element of innovation in this study is the production method, which combines extrusion, commonly used in the plastics industry, with hot flat pressing, a typical method used in the wood-based materials industry. This hybrid approach may effectively address the technological challenges associated with each process. Considering the research methods and the parameters investigated, although the conducted research primarily relates to wood–plastic composites, further investigation and analysis of the results obtained for these composites could also contribute to advancements in the plastics industry, even though this is not the primary focus of the study. It is expected to identify optimal formulations and production parameters for these composites. This approach aims to develop biocomposites with sufficient properties, promoting an environmental impact reduction.

## 2. Materials and Methods

### 2.1. Preparation of Biocomposites

**Figure 1:**
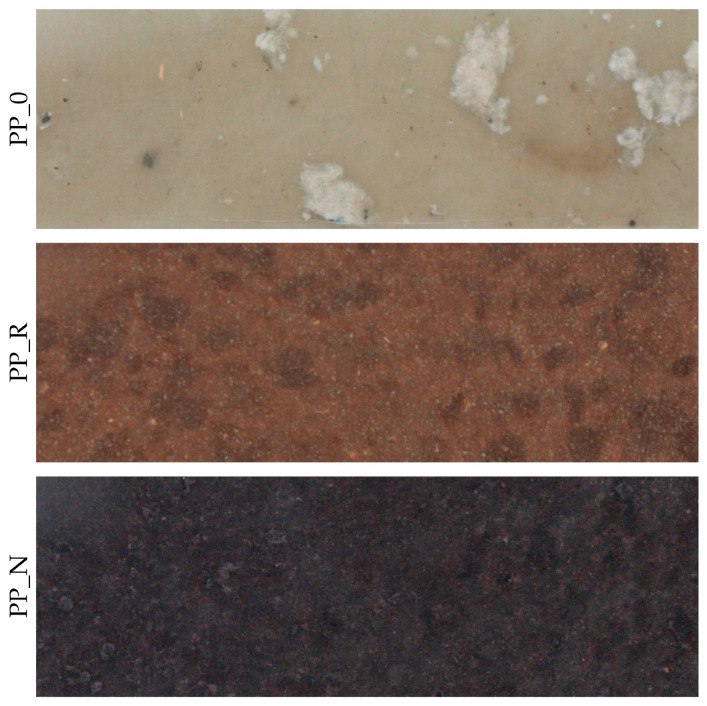
Examples of control and produced composites samples.

The composites were produced using oil-pressing residues from black cumin (*Nigella sativa*) and rapeseed (AL-PHADAR, Nienaszów, Poland) as fillers (separate variants), combined with a post-consumer polypropylene (PP) as a matrix. The manufacturing process methodology was described in the authors’ previous publications [17]. Initially, the plant residues were ground into fine particles with a size range of ∈<0.315–1.000> mm. The knife mill (OB-RPPD sp. z o.o., Czarna Woda, Poland) used for this operation was equipped with a sieve at the nozzle outlet with a mesh size of 1 mm and the container for the milled fraction with a 0.3 mm sieve separating the fine dust fraction. Particles larger than 1 mm and smaller than 0.3 mm were separated and rejected to standardize the range of fractions for all the fillers used. The post-consumer polypropylene used to form the composites was the residue from the production of the interfacing textile, milled in a knife mill (OB-RPPD sp. z o.o., Czarna Woda, Poland) and separated by a 6 mm mesh sieve. Fillers were mixed with polypropylene in a 1:1 mass ratio, using a high-speed mixer (KMOD SGGW, Warsaw, Poland). The blend was then processed using an extruder (Leistritz Extrusionstechnik GmbH, Nürnberg, Germany). The extruder had 6 heating zones with infinitely variable temperature control and double screws with a compound profile, L/D ratio 20:1. The material feeding was manual. During extrusion, the temperature in the individual sections of the extruder was maintained between 160 and 170 °C, and the screw speed was set to 16 RPM. After the extrusion process, the material was reduced in size using a knife mill (OB-RPPD sp. z o.o., Czarna Woda, Poland) to obtain particles suitable for the next processing stage. Composite boards with an assumed 900 kg/m^3^ density were manufactured from the previously extruded material. The board forms were made from 2.5 mm thick MDF with a rectangular cutout measuring 275 mm × 160 mm. Aluminum sheets and parchment paper were used to prevent the composite material from sticking to the frame. The material was hot-pressed using a single-shelf laboratory press (AB AK Eriksson, Mariannelund, Sweden) at 180 °C for the composites and 160 °C for the control samples. These temperatures were determined through technological trials and sensory evaluation. The presence of fillers resulted in a different heat transfer in the material, so it was necessary to use a higher temperature than in extrusion to properly heat the mat throughout. The initial pressing pressure was minimal, aiming only to close the press shelves and apply for one minute, after which the unit pressure (load) was increased to 0.5 MPa and after another minute to 1 MPa. The process was terminated after 5 min. After hot pressing, the boards were cooled for 5 min at 20 °C under minimal pressure using equipment from the Industrial Equipment Plant in Nysa, Poland. The material was left to season for 24 h, after which it was cut into 250 mm × 150 mm pieces and subsequently shaped into dimensions suitable for specific testing procedures. Examples of produced samples cut into 50 mm × 150 mm dimensions prepared for mechanical tests are presented in Figure 1.

The following codes were used to mark the obtained composites:

- PP_0: post-consumer polypropylene;
- PP_N: PP + nigella sativa pomace;
- PP_R: PP + rapeseed pomace.

### 2.2. Quality Parameters of Composites

Testing of the resulting composites was conducted following standards applied to wood-based materials, as the final stage of the production process involved hot flat pressing, a method typically used in the wood-based industry.

#### 2.2.1. Density and Mechanical Properties

The density of the prepared samples was determined according to the EN 323:1999 standard for wood-based panels [18]. Measurements were conducted using a PPS 1000.X2 electronic balance (Radwag, Radom, Poland) and an electronic caliper. The modulus of rupture (MOR) and modulus of elasticity (MOE) were tested in line with the EN 310:1994 standard for wood-based panels [19]. These tests were performed using a strength testing machine supplied by OB-RPPD sp. z o.o. (Czarna Woda, Poland), supported by OBRCzW_NET_MS version 1 software from the same manufacturer. The obtained values were expressed to three significant figures.

#### 2.2.2. Surface Roughness

Surface roughness measurements were carried out using a Surftest SJ-210 portable contact profilometer (Mitutoyo Co., Kawasaki, Japan), following the guidelines outlined in EN ISO 21920-2:2022-06 [20]. The parameters Rz, Ra, and Rq were evaluated for each composite variant and control sample.

#### 2.2.3. Contact Angle

The contact angle was measured using a Haas Phoenix 300 goniometer (Surface Eletro Optics, Suwon City, South Korea) with an automatic one μL droplet dispenser. Measurements were taken at 5, 20, 40, and 60 s after the droplet was placed on the sample surface. The contact angle was determined using an image analysis system (Image XP, Surface Electro Optics, version 5.8, Suwon City, South Korea), calculating the angle between the tangent to the droplet contour and a line intersecting its base.

#### 2.2.4. Water Absorption and Thickness Swelling

Thickness swelling (TS) after 2 h and 24 h of water immersion was measured in accordance with EN 317:1999 [21]. Water absorption after 2 h (WA_2_) and 24 h (WA_24_) was calculated using the following formula [22]:

WA_n_ = (m_n_ − m_0_)/m_0_ × 100%, (1)

where

m_0_—mass of sample, m_n_—mass of sample after soaking in water, n—time, n ∈ (2, 24).

#### 2.2.5. Fourier-Transform Infrared Spectroscopy (FTIR)

Infrared spectra were collected using a Cary 630 spectrometer (Agilent Technologies Inc., Santa Clara, CA, USA) with a diamond crystal ATR reflection accessory. Before measurements, a background spectrum was recorded. The measurements were performed over a wavelength range of 650–4000 cm^−1^ with a resolution of 4 cm^−1^ and 32 scans per spectrum with three scans for each sample. The analysis involved pressing the sample onto the crystal using a pressure clamp. Data were recorded using MicroLab FTIR software, version 5.7 (Agilent Technologies Inc., Santa Clara, CA, USA).

#### 2.2.6. Thermal Properties of Composites

Thermal stability was evaluated using a thermogravimetric analyzer (TGA/DSC 3+, Mettler Toledo, Greifensee, Switzerland). Approximately 7 mg of the ground material was placed in open 70 µL alumina crucibles and subjected to combustion in an oxygen atmosphere from 30 °C to 600 °C at a heating rate of 5 K/min, with a gas flow rate of 50 mL/min. Additionally, pyrolysis of the material was performed under a nitrogen atmosphere using the same temperature range, heating rate, and gas flow conditions. The TGA and DTG thermograms were processed using STARe software (version 16.20c, Mettler Toledo, Greifensee, Switzerland). The analysis was conducted in duplicate.

#### 2.2.7. SEM Analysis

Surface observations of the polypropylene control samples and the composites were carried out using a Phenom XL scanning electron microscope (Phenom World, Eindhoven, The Netherlands). To enhance conductivity, the samples were coated with a 5 nm layer of gold using an auto sputter coater (Leica EM ACE200; Leica Mikrosysteme GmbH, Vienna, Austria) before imaging. Photographs were captured at magnifications of 200×, 500× and 1000× and were recorded with Phenom ProSuite Software (version 5.4.7). The electron beam was operated at an acceleration voltage of 10 kV, with a chamber pressure of 10 Pa.

#### 2.2.8. Growth of Fungi

Square samples measuring 30 mm on each side were cut from the manufactured composites and control samples and then sterilized under UV light (Bionovo, Legnica, Poland) for 30 min. The sterile samples were placed on a maltose-agar medium containing 2.5% maltose extract (Linegal Chemicals sp. z o.o., Blizne Łaszczyńskiego, Poland) and 2.5% agar (Polaura, Morąg, Poland). To avoid direct contact between the biocomposites and the moist medium, plastic sterile pads were used. Fungal inocula, consisting of *Trichoderma viride* Pers., strain A-102 and *Chaetomium globosum* Kunze, and strain A-141 (ATCC 6205), sourced from the Department of Wood Science and Wood Preservation at Warsaw University of Life Sciences (Warsaw, Poland), were placed at four equidistant points around each disc, approximately 10 mm from the edge of the samples. The cultures were incubated for 14 days in a Thermolyne Type 42,000 incubator (ThermoFisher Scientific, Waltham, MA, USA) at 26 ± 2 °C and 63 ± 2% relative humidity. High-resolution images were taken at 24-h intervals using a laboratory photo-taking station. The assessment of fungal coverage on the sample surfaces followed the methods outlined in previous studies. The percentage of fungal coverage was calculated relative to the total surface area using ImageJ image analysis software (Fiji version 1.52i, Rasband WS. ImageJ, U.S. National Institutes of Health; Bethesda, MD, USA), with a precision of 5%. Each experimental variant was tested in triplicate. The percentage overgrowth of a sample of a particular variant was determined as the arithmetic mean of the values for the three samples.

### 2.3. Test Standards

The statistical analysis of the results was performed using Statistica version 13 (TIBCO Software Inc., CA, USA). The analysis of variance (ANOVA) was used to test (α = 0.05) for significant differences between variants. A comparison of the means was performed using Tukey’s test, with α = 0.05. To determine the properties of the produced composites, 10 repetitions were performed for each variant unless otherwise specified in the methodology of the specific experiment.

## 3. Results

### 3.1. Density and Mechanical Properties Results

Table 1 presents the results of the thickness measurements, while Table 2 shows the obtained density of prepared samples. Determining homogeneous groups indicates significant differences among tested variants. The control samples exhibited a thickness that exceeded the target values, resulting in a density lower than anticipated. This excess thickness and reduced density may suggest insufficient pressure during hot-pressing. Conversely, the composites demonstrated a thickness below the expected values, corresponding with a higher density than the control samples. The control samples consisted of polypropylene alone, so the bulk density of mats was higher than that of the variants of composites made of extruded polypropylene and lignocellulosic particles. This resulted in the differences in the height of mats during their manual forming. At the same material weight used for board production, the height of the mats was about 30% higher for the composites than for the polypropylene control samples. Therefore, the material flowed out sideways during the first stage of pressing—thickening and heating of the mats while closing the press shelves. These results suggest that forming the boards or the pressing parameters may require optimization to achieve the desired thickness and density.

**Figure 2:**
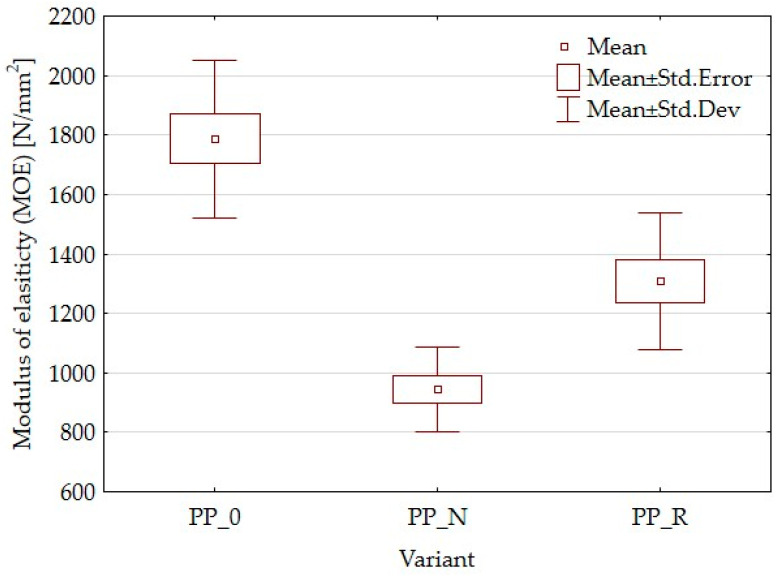
Box plot for modulus of elasticity of composites and control samples.

Figure 2 presents the results of the MOE tests for the different composite samples, highlighting the variations in mechanical performance based on the filler type. Both composites exhibited a reduction in MOE compared to the control sample. For the PP_R variant, the MOE decreased by 26.4%, from 1.78 GPa for the control sample to 1.31 GPa. Similarly, for the PP_N variant, the MOE declined by 46.9%, reaching 0.944 GPa compared to the polypropylene-only control.

The reduced MOE in the composites can be attributed to factors such as weak interfacial adhesion between the filler particles and the polymer matrix. This insufficient adhesion is likely due to the absence of coupling agents or modifiers, typically used to improve the interaction between polar filler materials and the non-polar polypropylene matrix. Consequently, localized weaknesses at the phase boundaries emerged, negatively impacting the mechanical performance of the composites.

**Figure 3:**
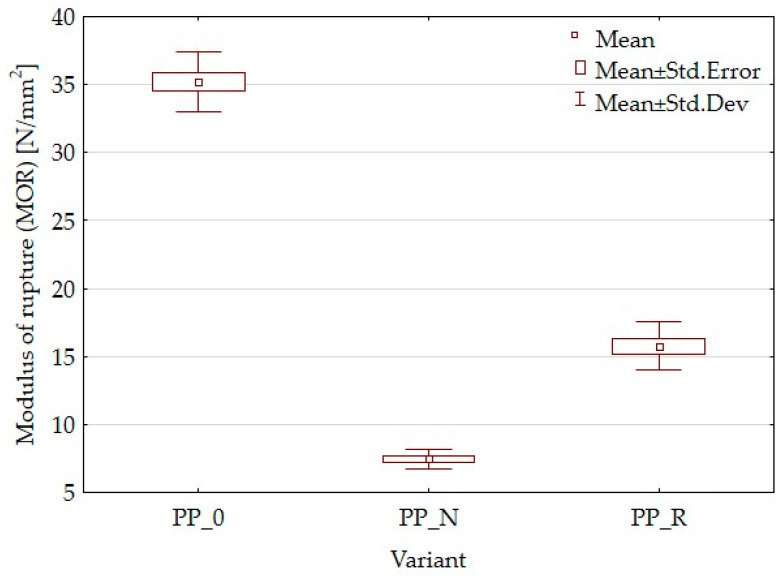
Box plot for modulus of rupture of composites and control samples.

Figure 3 presents the results of the modulus of rupture (MOR) tests for the composites and control samples. A significant decrease in MOR was observed in both composite variants compared to the control sample. For the PP_R variant, the MOR decreased by 55.1% from 35.2 MPa value for the control sample to 15.8 MPa. For the PP_N variant MOR decreased by 78.6% to 7.48 MPa value compared to the control sample made of polypropylene without fillers.

### 3.2. Surface Roughness Results

**Figure 4:**
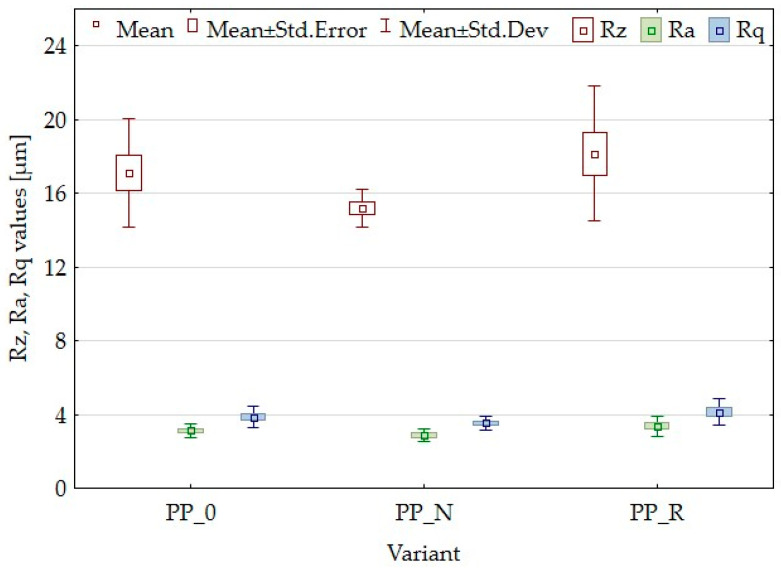
Box plot for Rz, Ra, and Rq parameters of composites and control samples.

Surface roughness parameters, including Rz, Ra, and Rq, are crucial for evaluating the texture and quality of a material’s surface. The surface structure largely depends on the manufacturing method and the molds used. For this study, it was important that uniform manufacturing conditions were maintained, allowing for a comparison of roughness parameters among the variants and control samples. The results of the surface roughness parameters illustrated in Figure 4 showed no significant differences in surface roughness among the various composite samples. However, it is worth noting that for the most sensitive parameter, Rz, the PP_N variant exhibited the most minor variation in results. This suggests that the nigella sativa filler contributed to a more consistent surface texture across the samples.

### 3.3. Contact Angle Results

**Figure 5:**
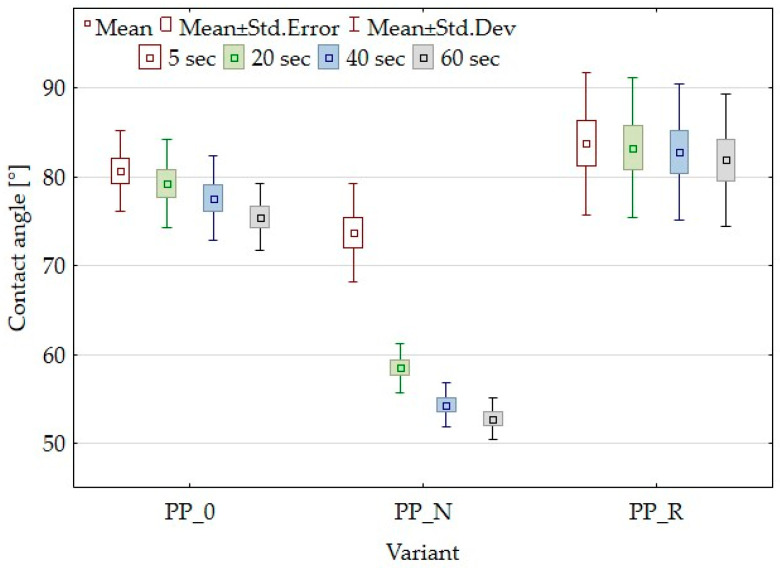
Box plot for contact angle of composites and control samples at 5, 20, 40, and 60 s.

The contact angle measurements of the composites were conducted to evaluate the wettability of the material’s surface, which is essential for understanding its adhesive and coating properties. Analysis of the results presented in Figure 5 revealed no significant differences in the contact angle values between the PP_0 and PP_R variants. In the case of the PP_N variant, the results at 5 s did not differ from those of the other variants. However, significant differences were observed at 20, 40, and 60 s, with a notable decrease in the contact angle for PP_N. This reduction indicates increased surface wettability. The differences in wettability can be attributed to the unique characteristics of the fillers used in the composites. In the case of PP_N, the increased wettability may result from the higher presence of hydrophilic hydroxyl (–OH) groups, as indicated by the FTIR spectra. These groups promote better adhesion between the water droplet and the composite surface, leading to enhanced droplet spreading. Additionally, the SEM images reveal the presence of microcracks on the surface of the PP_N composite, which may further influence wettability. These microcracks can act as capillaries, enabling the droplet to partially absorb into the material, amplifying the decrease in the contact angle.

### 3.4. Water Absorption and Thickness Swelling Results

Polypropylene (PP) is known for its hydrophobic nature [23], which provides resistance to water absorption and swelling [24]. The incorporation of lignocellulosic fillers alters this property, increasing the material’s affinity for water due to the hydrophilic characteristics of these fillers. Some water absorption was observed for the unfilled polypropylene sample (PP_0). This is likely due to the use of post-consumer PP, which may contain hydrophilic residues from prior use, contributing to water uptake.

**Figure 6:**
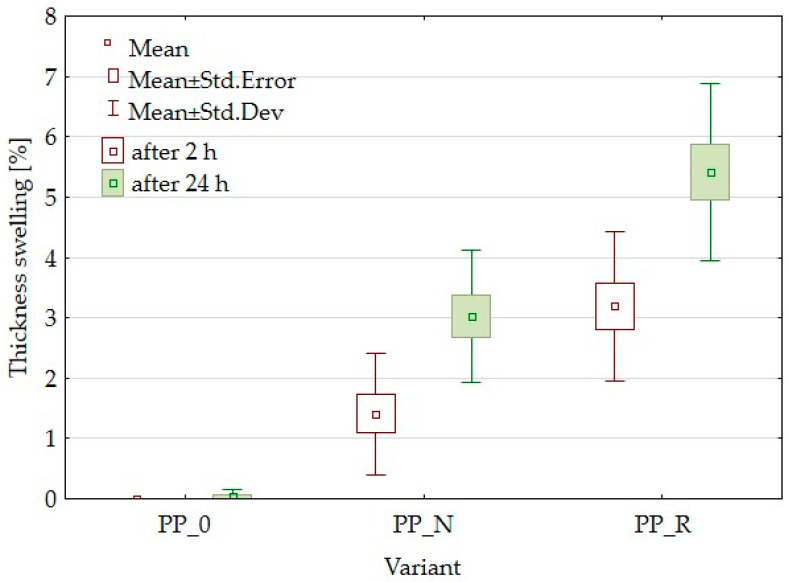
Box plot for thickness swelling after 2 h and 24 h soaking in water for composites and control samples.

**Figure 7:**
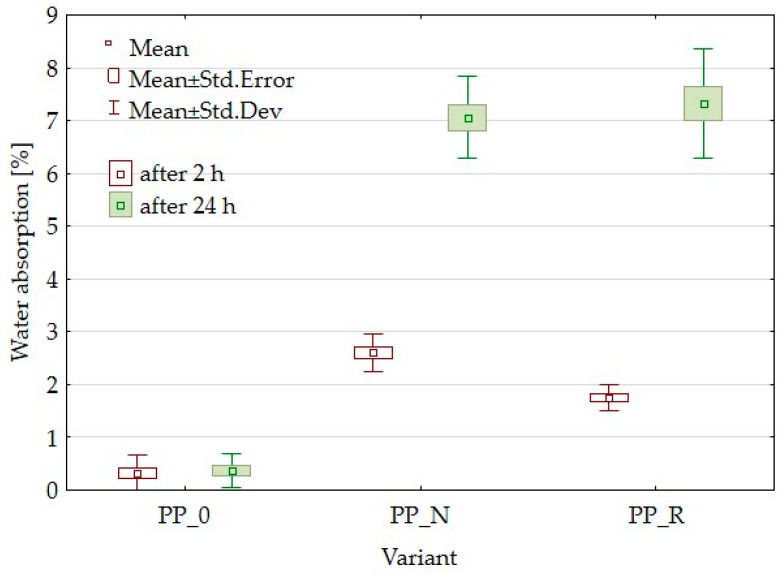
Box plot for water absorption after 2 h and 24 h soaking in water for composites and control samples.

Figure 6 presents the results of thickness swelling (TS), and Figure 7 the results of water absorption (WA) tests for the PP-based composites and control samples. As shown in Figure 6, the addition of black cumin pomace (PP_N) had a lesser impact on thickness swelling than rapeseed pomace (PP_R). After 24 h, the TS of PP_N reached a level comparable to PP_R after only 2 h of immersion. The water absorption data show that PP_N displayed higher WA after 2 h than PP_R, reaching similar levels after 24 h.

### 3.5. Fourier-Transform Infrared Spectroscopy (FTIR) Results

**Figure 8:**
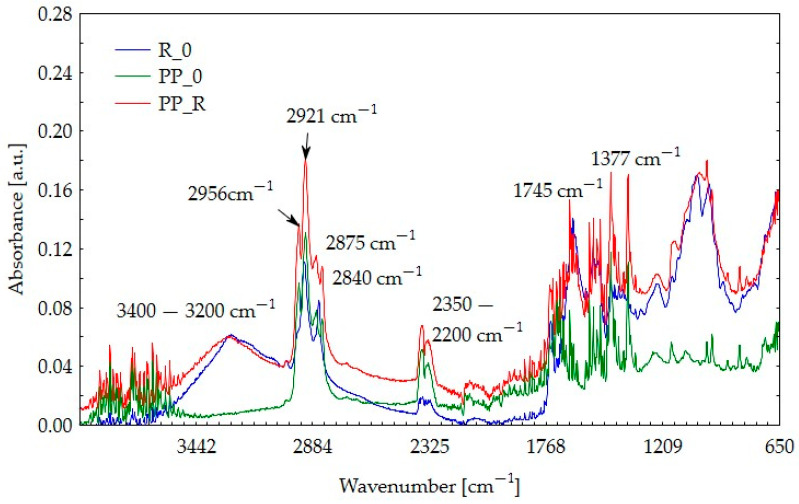
FTIR spectra for rapeseed pomace (R_0), pure polypropylene (PP_0), and PP_R composite.

**Figure 9:**
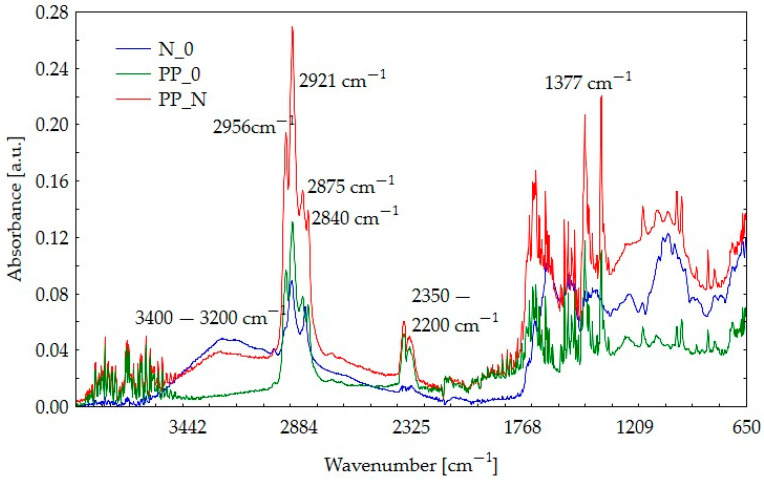
FTIR spectra for nigella pomace (N_0), pure polypropylene (PP_0), and PP_N composite.

Figure 8 and Figure 9 display the infrared absorbance spectra for pure polypropylene, raw pomace materials, and the resulting composite material. This layout allows for a comparative assessment of the changes in the material following composite production and provides insight into the distinct spectra of each component. The materials are labeled as follows:

For this analysis, raw materials were marked with the following codes:

- R_0: rapeseed seed pomace;
- N_0: sunflower seed pomace.

Characteristic of polypropylene are peaks at 2956 cm^−1^, 2921 cm^−1^, 2875 cm^−1^, and 2840 cm^−1^, corresponding to C–H stretching vibrations, which are visible at PP_0 spectra, which also peak at 1377 cm^−1^ representing C–H deformation [25]. Due to the non-reactive nature of PP [26], these peaks remain visible in the composite spectra. For the research, post-consumer polypropylene was used, which is why, in addition to the characteristic peaks, other peaks can also be observed due to the presence of numerous impurities in the material. The broad peak around 3200–3400 cm^−1^ is attributed to O–H stretching in hydroxyl groups present in cellulose and hemicellulose, typical of lignocellulosic materials [27,28] especially since this band is followed by the presence of spectra at frequencies 1600–1300 cm^−1^, 1200–1000 cm^−1^, and 800–600 cm^−1^ [29]. Peaks around 3200–3400 cm^−1^ may also be connected to the moisture content. The decrease of this peak in the PP_N composite could be due to water vapor during composite production, but we do not observe this in the case of PP_R. The peaks around 2200–2350 cm^−1^ in all samples may be associated with the presence of carbon dioxide (CO_2_) in the measurement environment and may be treated as spectra disturbance [30]. Peaks around 1734–1738 cm^−1^ correspond to C=O vibrations in hemicellulose [28] but can also represent esters and ketones [31]. It is also characteristic for lignin (aromatic structures) in the region 1550–1300 cm^−1^ and for the cellulose in 1160–900 cm^−1^ that peaks are visible [32]. Analysis of both composite spectra showed that none of the characteristic peaks were reduced, so it may be assumed that no component degradation occurred. When comparing rapeseed and blackseed oil spectra from available publications to composites spectra, we can distinguish peaks connected to pomace residual oils [33,34]. It can be observed that the peak of about 1745 cm^−1^, known as the ester peak [32], is relatively sharp in the case of the R_0 composite compared to that in N_0.

### 3.6. Results of Thermal Properties of Composites

**Figure 10:**
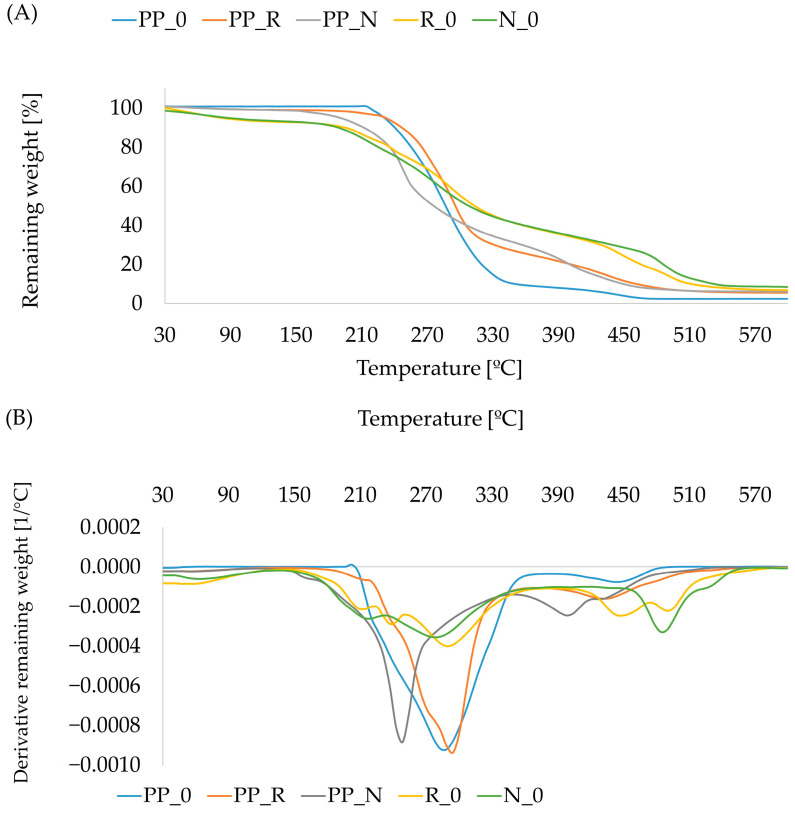
Thermogravimetric curves TGA (**A**) and DTG (**B**) for combustion in oxygen for composites and control samples.

**Figure 11:**
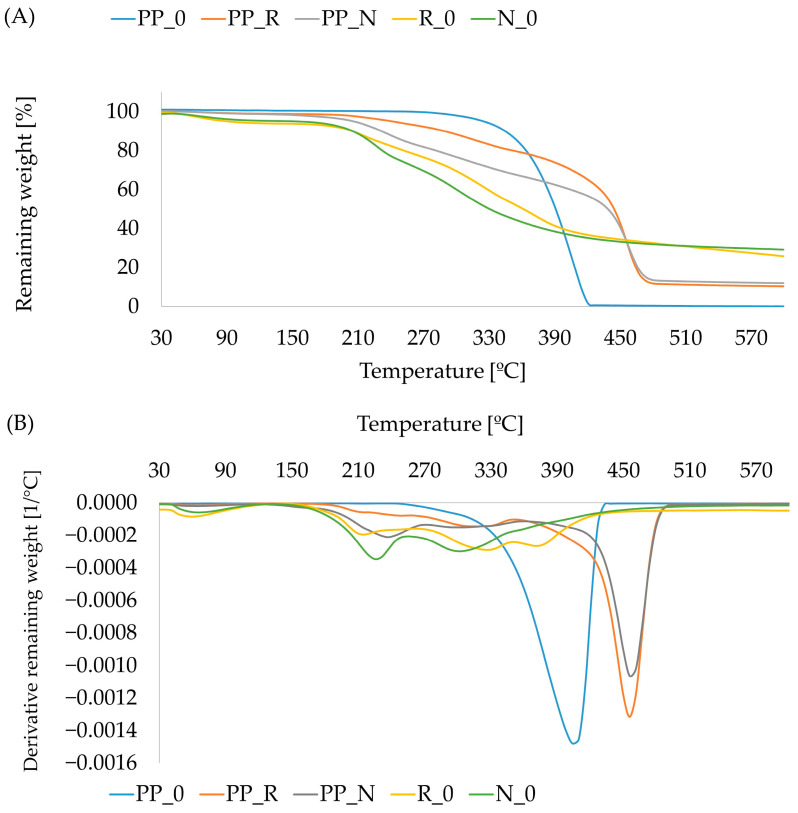
Thermogravimetric curves TGA (**A**) and DTG (**B**) for heating in nitrogen atmosphere for composites and control samples.

The TGA analysis presents mass changes of the tested sample when temperature increases within a controlled environment. This offers valuable information about thermal stability or material composition. The findings from the tests conducted in oxygen are illustrated in Figure 10, while the results from tests in nitrogen are shown in Figure 11. According to the literature, the softening temperature of polypropylene (PP) is approximately 160 °C [35], while the processing temperature falls between 235 and 250 °C. At these temperatures, no degradation should be observed. However, in our control sample, the mass loss began at around 215 °C, likely due to the presence of residues in post-consumer polypropylene. Polypropylene decomposes at a temperature range of 320–400 °C [20]; in our sample, in this area, we can observe secondary weight loss.

In the case of lignocellulosic materials, the thermal characteristics depend significantly on the proportions of individual components, such as lignin, cellulose, and hemicellulose. The degradation temperature of hemicellulose is mainly between 150 and 350 °C, whereas, for cellulose, it is between 275 and 350 °C, and, for lignin, it is between 250 and 500 °C [36,37]. The early mass loss of pomace (R_0 and N_0) during combustion can be observed due to the evaporation of volatile organic compounds (VOCs). These components are abundant in pomace due to the presence of residual oils or organic acids [38,39]. In the temperature range of 180–320 °C, R_0 exhibited a smaller mass loss compared to N_0. At a later stage, within the 420–550 °C range, the mass loss for N_0 occurred at a slower rate. Ultimately, the residual mass was 6.7% for R_0 and 8.4% for N_0. In the case of produced composites, mass loss began at around 150 °C, with a minor earlier loss attributed to water evaporation. We did not observe mass loss connected with volatile compounds at the beginning of the process, as they were removed during extrusion and the flat pressing process. For the PP_N composite, the degradation process occurred more rapidly, with a DTG curve peak at approximately 250 °C and a second peak at around 390 °C. Aside from the initial phase, the TGA curve for the PP_R composite remained above that of the reference PP_0 sample. The last stage of decomposition was at the range of 390–510 °C and is related to char forming [40]. According to the literature, blackseed oil degradation is observed between 290–420 °C, similar to rapeseed oil [37]. Due to this, the oil content in pomace secondary peaks can be observed.

In a nitrogen atmosphere, mass loss begins at a temperature similar to that observed in oxygen. However, the process proceeds more steadily due to the lack of oxidation effects. In the temperature range of 210–410 °C, the mass loss for N_0 occurred more rapidly compared to R_0. At the end of the pyrolysis process, the residual mass was 25.7% for R_0 and 29.1% for N_0. The TGA profiles of composites corresponded to the behavior observed in the raw materials. Within the degradation range of 160–450 °C, the PP_N composites exhibited a greater mass loss compared to the PP_R composites. The relationship was delayed at the final stage along the temperature axis due to the presence of polypropylene, for which mass loss begins at a later stage. We did not observe mass loss connected with volatile compounds at the beginning of the process, as they were removed during extrusion and the flat pressing process. Notably, in the PP_N composite, a small initial peak appeared at around 250 °C. The final decomposition of PP_0 occurred at around 420 °C, whereas for the composites, this process was delayed until approximately 480 °C, leaving a residual mass of about 10%.

### 3.7. Results of SEM Analysis

**Figure 12:**
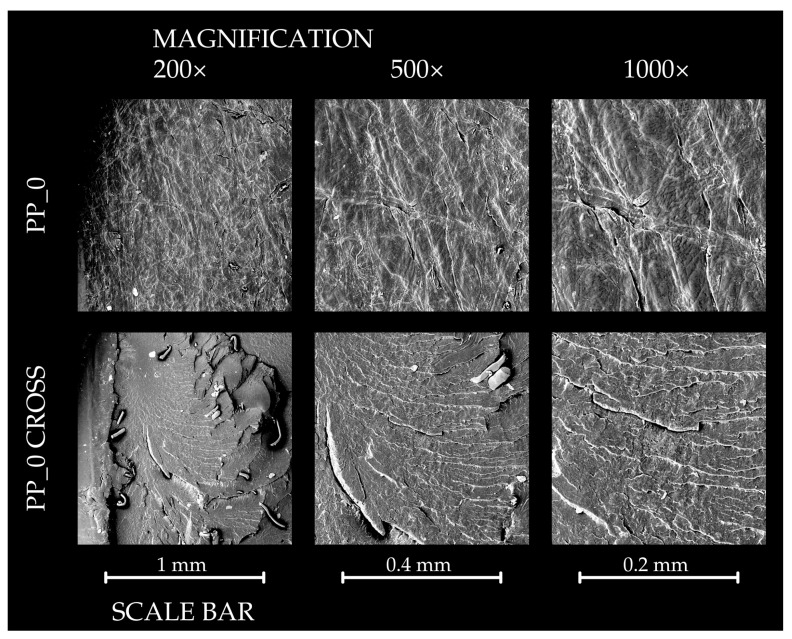
The SEM results of the surface observation of control pure polypropylene sample—surface and cross section.

**Figure 13:**
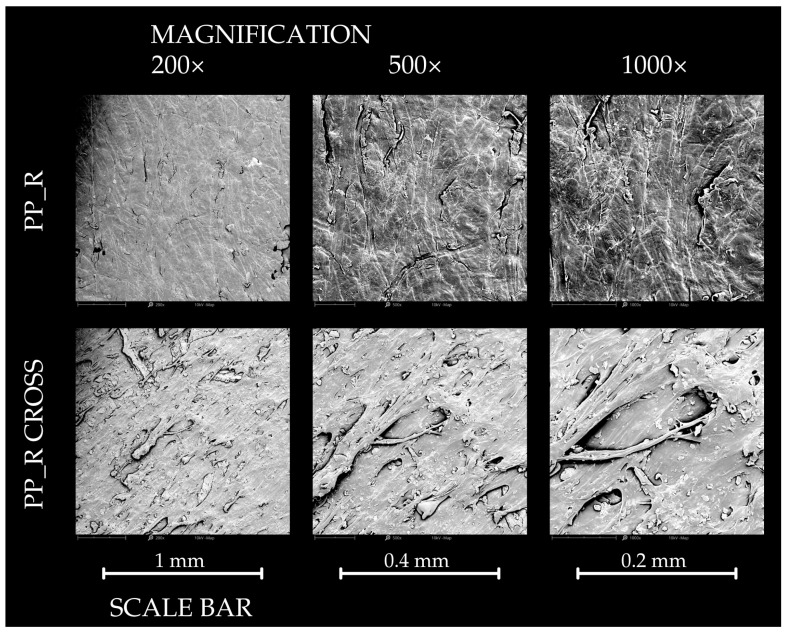
The SEM results of the surface observation of PP_R sample—surface and cross section.

**Figure 14:**
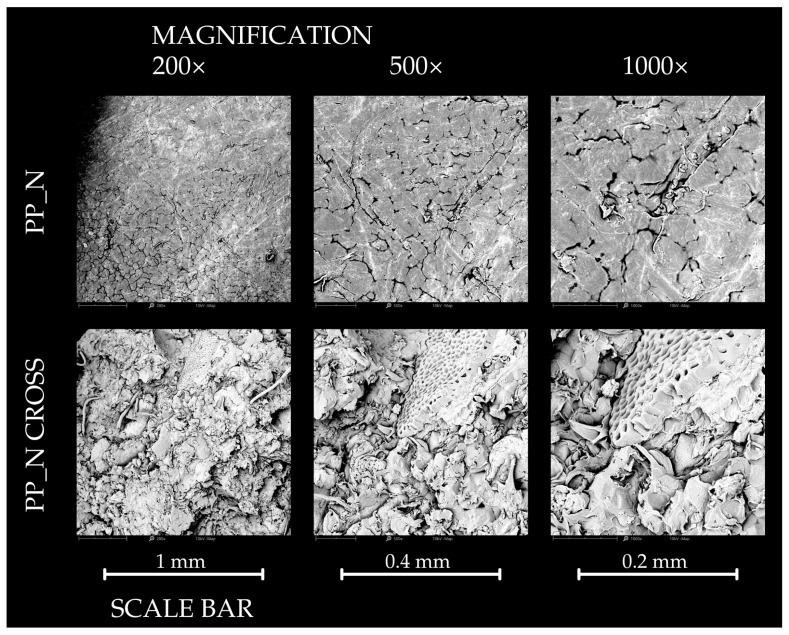
The SEM results of the surface observation of PP_N sample—surface and cross section.

The results of the SEM observations of the surface and cross-section of pure polypropylene samples and composites are presented in Figure 12, Figure 13 and Figure 14. The PP_0 samples exhibited a relatively smooth surface with a visible structure attributed to the production process. Cross-sectional views revealed minor impurities, but the overall structure remained compact. For the PP_R composites, the surface structure was similar to that of PP_0. However, the cross-section showed more irregularities due to the presence of lignocellulosic particles. Despite these variations, the structure appeared cohesive, with only small voids observable. In contrast, the PP_N composite was characterized by an irregular surface with visible empty spaces and visible filler particles. Its cross-section differed significantly from PP_R, displaying a less compact structure with prominent clusters of filler particles in comparison to other samples.

### 3.8. Growth of Fungi Results

**Figure 15:**
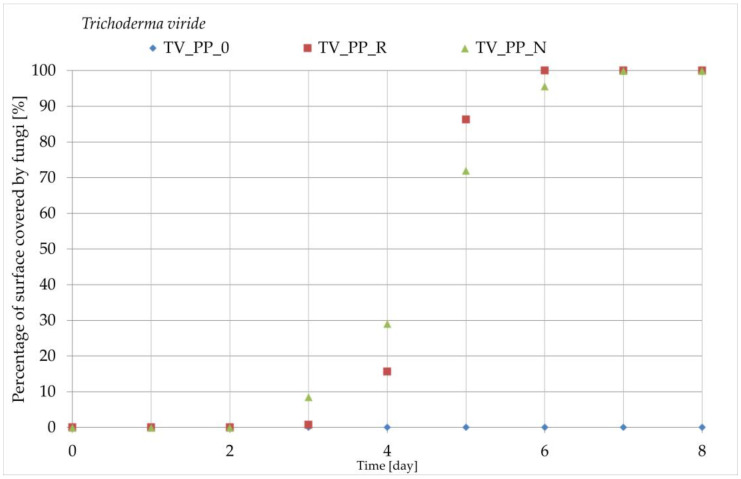
Results of the growth of *Trichoderma viride* on the surface of composites and control samples.

**Figure 16:**
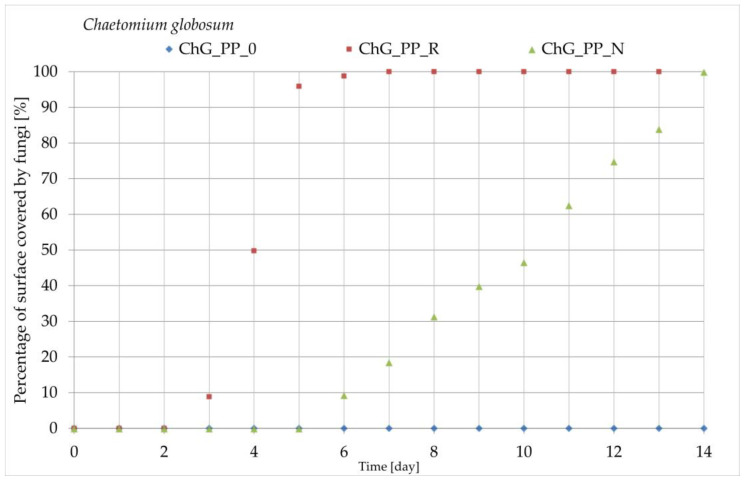
Results of the growth of *Chaetomium globosum* on the surface of composites and control samples.

**Figure 17:**
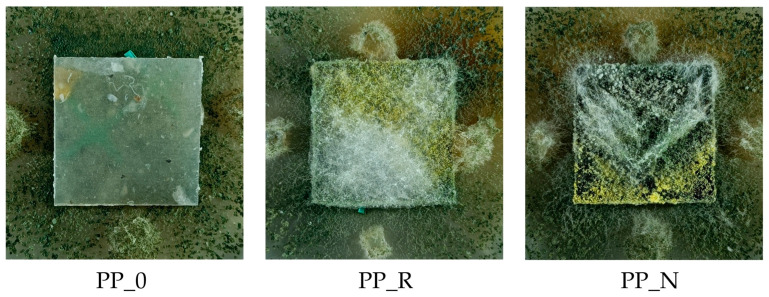
Example of mold growth (*Trichoderma viride*) after 8 days of exposure—control sample and composites.

**Figure 18:**
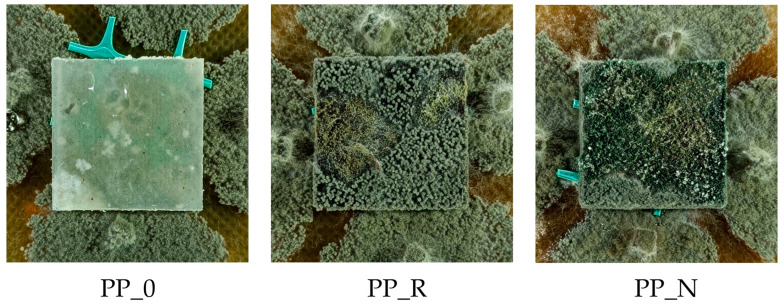
Example of mold growth (*Chaetomium globosum*) after 14 days of exposure—control sample and composites.

The results of the degree of surface overgrowth by fungi are presented in Figure 15 and Figure 16. Additionally, Figure 17 shows images of samples overgrown by Trichoderma viride, and Figure 18 displays images for *Chaetomium globosum*. For both fungi, no surface overgrowth was observed on the control polypropylene samples (PP_0) despite minor impurities present in the material. However, notable differences emerged among the composite samples. In the case of *Trichoderma viride*, fungal growth began on the PP_N composite on the second day, reaching full coverage (100%) by day 7. For the PP_R composite, fungal growth was observed starting on day 3, achieving nearly complete overgrowth by day 6. Regarding *Chaetomium globosum*, colonization on the PP_N sample also began on the second day, with full coverage achieved by day 7. However, on the PP_R composite, growth initiation started on day 5, progressing more gradually and reaching complete overgrowth only by day 14.

## 4. Discussion

Lignocellulosic fillers are applied to enhance the mechanical properties of polymer matrices when used as reinforcing agents. These fillers contribute to the composite’s stiffness and help distribute applied stress more effectively, potentially increasing the modulus of elasticity (MOE) [26,41]. The effectiveness of reinforcement can vary based on specific formulations and processing conditions. In this study, both MOE and MOR parameters were reduced. The composites demonstrated higher elasticity than the control samples and were less durable. Kaya et al. [36] obtained PP composites with olive pomace filler with an MOE parameter value higher than the PP sample, increasing the filler amount starting from 0% to 40% of the pomace amount. However, it is worth noting that the modulus for the reference sample was lower than that in the current article, while the value obtained with 40% filler was comparable to that for PP_R (50% filler). Berger et al. [42] studied PP composites with pine and grape pomace particles, observing an increase in MOE and a decrease in MOR. The reduction of mechanical parameters is often attributed to inadequate adhesion between the particles and the polymer matrix, which results in weak interfacial bonding. The differences in the parameters of the individual variants may result from particle geometry or size or the content of residual oils. This suggests that it would be appropriate to expand the research to include the influence of particle size or to conduct detailed chemical studies focusing on adhesion and in later stages, consider the pretreatment of fillers. In addition to insufficient particle adhesion, it is important to consider the density parameters and contemplate setting a higher target density for the composite. To achieve this, increasing the material weight for pressing will be necessary, which will result in a greater mat height. This will require careful control of the pressing process to avoid material outflow. SEM observations for the PP_N composite revealed noticeable gaps between the lignocellulosic particles and the polypropylene matrix. These gaps likely contribute to the reduced mechanical strength. These voids may also explain why PP_N exhibited lower thickness swelling than PP_R. The gaps potentially allowed for water uptake without extensive expansion of sample thickness, even with the same water absorption as in the case of PP_R. Surface roughness measurements indicated no significant differences across the composite variants, which was a positive outcome suggesting that the particle addition does not detract from the surface quality.

Contact angle measurements allowed for an assessment of how particle addition affects surface wettability. While the PP_R composite did not show a noticeable change in the contact angle compared to the control (PP_0), PP_N demonstrated differences in wettability. The low variation in roughness parameter R_z_ for PP_N may have also contributed to the contact angle effect. Improved PP_N wettability may also be the result of the increased presence of hydrophilic hydroxyl (–OH) groups and microcracks on the surface. This suggests that the composition of the filler can be tailored to modify surface wettability.

FTIR analysis showed no significant changes in the components. However, differences in peak intensities were observed between the two fillers (black cumin and rapeseed). This suggests variations in the composition and proportions of components such as hemicellulose, cellulose, lignin, and residual oils. This difference was further supported by TGA analysis, where PP_N displayed a more rapid mass loss, potentially linked to a higher proportion of particles that decompose more readily.

The fungal growth tests on the composites revealed that the addition of lignocellulosic particles increased susceptibility to fungal colonization. This is a common occurrence when using organic fillers. However, the extent of fungal growth varied between the PP_R and PP_N composites, suggesting the type of pomace used influences the material’s resistance to biological degradation.

## 5. Conclusions

This study highlights the potential of different types of pomace as promising fillers in polypropylene composites, influencing physical, mechanical, thermal, and biological properties. Although the composites exhibited lower mechanical strength and reduced thermal stability, it was noteworthy that their surface roughness parameters remained stable. Additionally, the use of black cumin pomace enhanced surface wettability. Furthermore, the addition of lignocellulosic particles introduced a susceptibility to fungal growth, which may serve as an indicator of the composites’ potential for biodegradability. From an economic perspective, the use of oilseed pomace in the post-consumer polypropylene matrix is advantageous due to its typically lower cost compared to primary raw materials. Additionally, processing this by-product helps reduce the expenses related to its storage and disposal, making the production processes more cost-effective and environmentally sustainable. However, further optimization of composite manufacturing processes and material composition is needed to improve mechanical properties and expand the application potential of these composites.

## Figures and Tables

**Table 1:** Thickness of composites and control samples.

Variant	Average Thickness [mm]	Coefficient of Variation [%]
PP_0	2.56 ^a^	3.81
PP_N	2.45 ^b^	2.79
PE_R	2.35 ^c^	1.69

^a, b, c^ Homogenous groups by using Tukey Test.

**Table 2:** Density of composites and control samples.

Variant	Average Density [kg/m^3^]	Coefficient of Variation [%]
PP_0	853 ^a^	2.46
PP_N	942 ^b^	2.70
PE_R	993 ^c^	1.51

^a, b, c^ Homogenous groups by using Tukey Test.

## Data Availability

The original contributions presented in the study are included in the article; further inquiries can be directed to the corresponding author.
